# Microclimate Performance Analysis of Urban Vegetation: Evidence from Hot Humid Middle Eastern Cities

**DOI:** 10.3390/plants14040521

**Published:** 2025-02-08

**Authors:** Sarah Al-Hajri, Baqer Al-Ramadan, Md Shafiullah, Syed Masiur Rahman

**Affiliations:** 1 Architecture & City Design Department, King Fahd University of Petroleum & Minerals, Dhahran 31261, Saudi Arabia; g202201360@kfupm.edu.sa; 2 Interdisciplinary Research Center for Smart Mobility & Logistics, King Fahd University of Petroleum & Minerals, Dhahran 31261, Saudi Arabia; 3 Control & Instrumentation Engineering Department, King Fahd University of Petroleum & Minerals, Dhahran 31261, Saudi Arabia; shafiullah@kfupm.edu.sa; 4 Interdisciplinary Research Center for Sustainable Energy Systems, King Fahd University of Petroleum & Minerals, Dhahran 31261, Saudi Arabia; 5 Department of Civil and Environmental Engineering, King Fahd University of Petroleum & Minerals, Dhahran 31261, Saudi Arabia; smrahman@kfupm.edu.sa; 6 Interdisciplinary Research Center for Construction and Building Materials, King Fahd University of Petroleum & Minerals, Dhahran 31261, Saudi Arabia; 7 Applied Research Center for Environment and Marine Studies (ARC-EMS), King Fahd University of Petroleum & Minerals, Dhahran 31261, Saudi Arabia

**Keywords:** urban heat island (UHI), geographic information systems (GIS), vegetation strategies, sustainable urban planning, thermal comfort, urban microclimate, green infrastructure, spatial analysis, environmental sustainability, hot and humid climates

## Abstract

Urban heat islands (UHIs) pose a growing challenge in rapidly urbanizing areas, necessitating effective mitigation strategies to enhance environmental sustainability and human well-being. This study examined the role of vegetation in regulating urban microclimates, focusing on its ability to mitigate the effects of UHIs, promote thermal comfort, and enhance urban esthetics. The study drew on existing research that employed spatial analysis and Geographic Information Systems (GIS) to explore the relationship between vegetation metrics and reductions in surface temperature. Municipal initiatives in Khobar, Saudi Arabia, including tree-planting programs and street humanization projects, aimed to improve urban esthetics and pedestrian experiences. Although these efforts enhanced urban livability, they lacked a comprehensive ecological perspective, emphasizing the need for strategies that integrate thermal comfort, environmental resilience, and broader sustainability goals. The analysis demonstrated the societal and environmental benefits of tree-planting activities and linked urban vegetation plans to the achievement of Sustainable Development Goals (SDGs). The results highlighted the importance of incorporating green infrastructure in urban development to mitigate the effects of UHIs, improve air quality, and enhance overall urban livability. This paper proposed a framework for sustainable urban design, offering practical insights for policymakers and urban planners working to create resilient, environmentally conscious communities in extreme climates.

## 1. Introduction

Rapid urbanization and climate change have exacerbated the urban heat island (UHI) effect, particularly in hot, humid cities, where extreme temperatures pose significant challenges to urban sustainability and livability. While the mitigating effects of vegetation on these phenomena are increasingly recognized, there remains a significant gap in synthesizing research on how different types and densities of vegetation directly influence urban microclimates [1].

A prominent example of urban greening initiatives is the “Green Riyadh” project, recognized as one of the largest afforestation efforts globally. This initiative aligns with the Kingdom of Saudi Arabia’s Vision 2030 objectives to promote sustainable development and elevate Riyadh’s status among global cities [2]. Companies such as Dar and Emaar have spearheaded several projects to create eco-friendly residential communities. Notably, with a dedicated budget of 20 million Saudi riyals for reforestation in the “Saraya Al Narjis” community, these efforts reflect a strong commitment to social responsibility and community development [2,3]. The primary goal of this initiative is to enhance the quality of life for residents while fostering an ecologically sound environment that supports biodiversity and addresses climate change, ensuring the preservation of natural resources for future generations.

The “Green Riyadh” project is recognized as the most extensive global urban afforestation initiative, aiming to increase per capita green space and enhance vegetation coverage through intensive reforestation efforts. Such initiatives are crucial for mitigating rising urban temperatures and improving city microclimates. Several review articles in urban environmental studies have significantly advanced the understanding of UHI challenges and efforts to enhance thermal comfort in densely populated areas [4]. Research comparing UHI mitigation measures has focused on sustainable urban development [5]. Studies have thoroughly examined various types of green infrastructure, highlighting their potential to reduce urban heat through cooling effects and improved thermal comfort. Green infrastructure, including urban parks, urban forests, street trees, green roofs, and green walls, is essential for achieving ecological, economic, and social benefits that contribute to sustainable urban development and climate adaptation [6].

A study assessed the use of urban green infrastructure to improve thermal comfort in densely populated metropolitan areas, providing valuable insights into current research trends and implementation strategies [7]. While the studies mentioned above made significant contributions, several topics remain underexplored in the literature. One area that lacks sufficient focus is the integration of urban forestry and agroforestry techniques to reduce urban heat islands and improve microclimate conditions. Although there have been notable contributions in this field, the integration of urban forestry and agroforestry to mitigate UHIs and enhance microclimates remains limited. Moreover, the need for more quantitative assessments of how street vegetation affects air quality, particularly the dispersion of particulate matter in urban environments, has been emphasized [8].

This review synthesizes the existing literature on the relationship between vegetation indicators and urban microclimates, with particular emphasis on extreme weather conditions. By consolidating findings from multiple studies, this paper aims to provide actionable insights for urban planners and policymakers, emphasizing the role of green infrastructure in mitigating UHIs and promoting sustainable urban development. This synthesis will offer valuable information to inform practical recommendations for urban planning and design, focusing on the deliberate integration of green spaces to enhance environmental quality, resilience, and overall well-being in urban communities.

This paper systematically reviews the literature on urban microclimates, vegetation dynamics, and urban tree canopy coverage, examining their relationships with temperature, humidity, and vegetation metrics. Using GIS technology, the study modeled and analyzed urban microclimate data while exploring the esthetic benefits of vegetation. The research investigated heat reduction methods associated with urban design decisions, including street orientation, tree species selection, sky view factors, and overall street design. The study also emphasized the broader implications of sustainable community planning by correlating findings with the Sustainable Development Goals (SDGs). The Urban Planning and Design Considerations Framework provides practical suggestions for urban planners and designers using GIS technology. The conclusion synthesizes key findings, discusses practical implications, and proposes future research directions. This systematic approach thoroughly examined the complex relationships between urban vegetation, microclimates, and sustainable urban growth.

## 2. Materials and Methods

**Figure 1:**
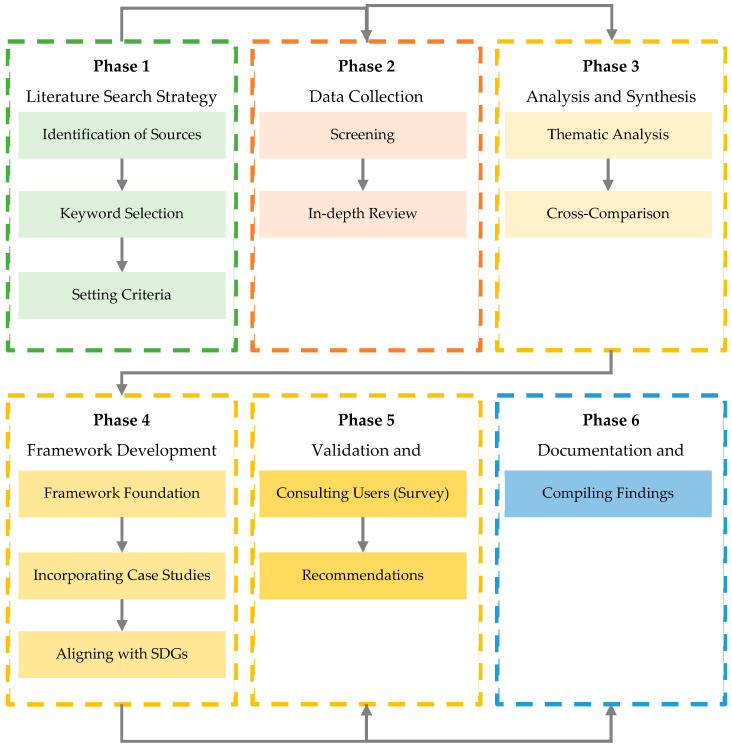
Adopted review methodology.

A structured approach was used to systematically collect, analyze, and synthesize current knowledge on the crucial role of vegetation in mitigating UHI and enhancing urban thermal comfort. This process involved a comprehensive search across various scholarly databases (i.e., Scopus, Web of Science, and Google Scholar), using a combination of appropriate search terms to ensure the inclusion of significant and up-to-date research in urban environmental science and planning. The selection criteria focused on urban microclimates, the specific effects of vegetation, and the practical applicability of the findings for urban planning and design. The methodological process was divided into six phases (Figure 1), ranging from literature review and data collection to analysis and documentation.

The research began with a literature search strategy, identifying key databases and sources within urban planning, environmental science, and geography. This phase was essential for gathering diverse scholarly journals and credible research contributions. Search terms such as “urban microclimate”, “vegetation”, and “urban heat island” were used to filter relevant information. Inclusion and exclusion criteria were established to ensure the relevance of the selected studies, with a focus on recent publications. The first step involved gathering data through reviewing abstracts and papers discussing vegetation’s impact on urban microclimates. A detailed analysis of specific articles provided valuable insights into the research methods, conclusions, and the complex role of vegetation in urban environmental management.

The analysis and synthesis process involved organizing information into logical themes using thematic analysis. A cross-comparison of studies was necessary to identify trends, contradictions, and gaps in the literature. An urban planning and design considerations framework was developed, incorporating case studies that highlighted effective strategies and interventions. This framework aligned with the SDGs and emphasized the role of vegetation in advancing urban sustainability. Literature research and bibliometric analyses were conducted using Scopus and Web of Science, ensuring access to high-quality, peer-reviewed articles. VOSviewer 1.6.20 software was employed for bibliometric mapping and network visualization, providing insights into research trends and collaborations. These tools collectively supported a comprehensive and transparent approach to synthesizing the findings and constructing a conceptual framework, ensuring the research’s methodological rigor and repeatability.

### Bibliometric Analysis: Vegetation Coverage Impact on Urban Microclimates

**Figure 2:**
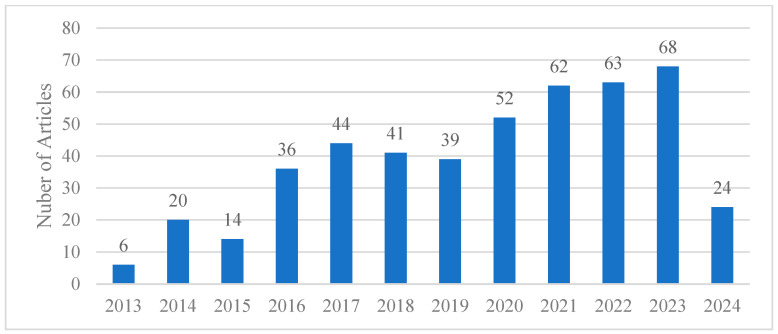
Publications per year with search keywords: (“vegetation” OR “tree” OR “plant”) AND (“microclimate”) AND (“Urban Heat Island” OR “UHI”).

A bibliometric methodology was employed to examine the effects of vegetation on urban microclimates, detailing the criteria for literature selection and the bibliometric analysis that was conducted. When the search included keywords such as “vegetation”, “tree”, and “plant” combined with “microclimate” and “Urban Heat Island” (UHI), as shown in Figure 2, the results were broader, reflecting the prevalence of studies that did not use remote sensing technologies, particularly GIS. However, integrating GIS into the search terms produced fewer results, indicating that the application of GIS in this field is relatively new.

**Figure 3:**
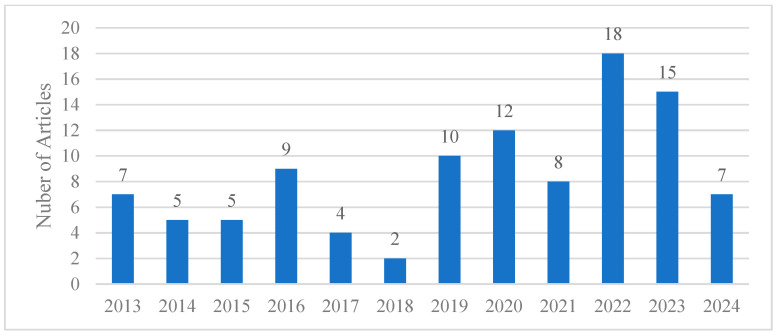
Publications per year with search keywords: (“vegetation” OR “tree” OR “plant”) AND (“microclimate”) AND (“GIS” OR “Geographic Information System”).

The search strategy used for this review incorporated keywords like “vegetation”, “tree”, and “plant”, combined with “microclimate” and either “GIS” or “Geographic Information System” (Figure 3). This approach initiated secondary data collection, focusing on sourcing articles essential for evaluating the growing scholarly interest in this domain. The bibliometric analysis revealed a noticeable trend in research publications related to integrating green spaces into urban planning. The timeline of publications from 2013 to 2023 showed a consistent increase, with a marked rise beginning in 2020 and peaking in 2022. This trend reflects an increasing recognition of the role of vegetation in fostering sustainable urban environments. The data extracted from these publications provided a critical foundation for a comprehensive review of urban climate mitigation strategies and their implications for contemporary urban landscapes.

**Figure 4:**
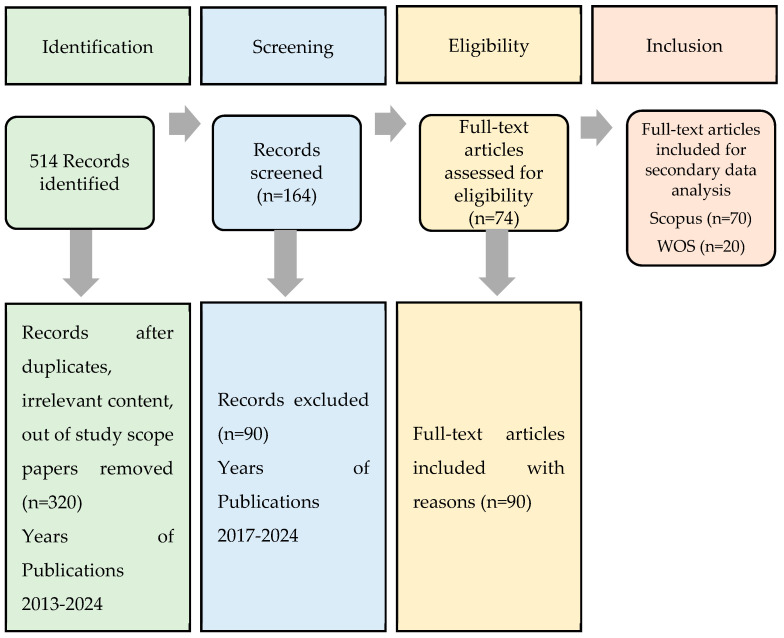
PRISMA flow diagram: included articles and keywords.

This section outlines a systematic approach to addressing the research gaps in UHI mitigation strategies, establishing a detailed framework that extends beyond urban layout to include improvements in air quality, natural airflow, and occupant well-being. The study also emphasizes the ongoing need for further research to improve thermal comfort and enhance the visual appeal of urban environments. To refine the review’s focus, search keywords were carefully adjusted to align more closely with the study’s scope, targeting the literature specifically relevant to the climatic aspects of urban design. As part of this refined approach, the PRISMA (Preferred Reporting Items for Systematic Reviews and Meta-Analyses) flow diagram was employed (Figure 4). This diagram provides a clear, concise visual representation of the systematic process used to identify, screen, and select studies for inclusion, ensuring transparency and replicability in the research methodology [9].

## 3. Results and Discussions

**Figure 5:**
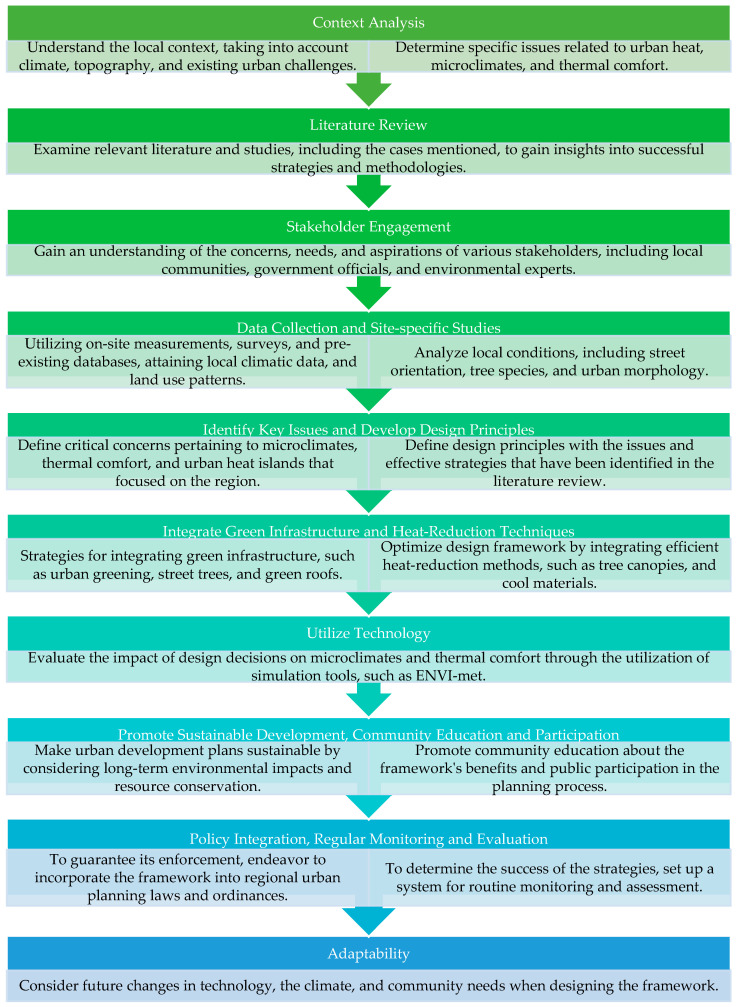
Urban planning and design framework.

Figure 5 illustrates the framework used in this study to establish vegetation guidelines for streets, detailing a step-by-step process that integrates contextual analysis, data-driven insights, and stakeholder engagement to develop sustainable urban solutions. The framework begins with an understanding of the local context and urban challenges, followed by a comprehensive literature review to gather evidence-based strategies. It incorporates stakeholder input to address community-specific needs and employs advanced tools such as GIS and simulations to inform decision-making.

By focusing on green infrastructure integration, sustainable development, and adaptability, this framework provides a systematic approach to creating climate-resilient, thermally comfortable, and environmentally sustainable urban streetscapes. It considers multiple factors, including climate and urban design, and offers an organized method for selecting appropriate plants to enhance visual appeal, air quality, and pedestrian shade. This study examined the role of vegetation in shaping urban microclimates and its impact on the thermal, esthetic, and ecological aspects of cities. It highlighted vegetation’s role in mitigating UHI effects through natural processes like evapotranspiration and surface cooling. The study emphasized the use of GIS and spatial analysis tools in mapping and predicting microclimatic outcomes, aiding effective urban planning. Additionally, it discussed vegetation’s cooling effects, energy conservation benefits, and contributions to climate resilience. Beyond its climatic advantages, vegetation improves urban esthetics, promoting mental health, increasing property values, and enhancing recreational spaces, and thus supporting the creation of sustainable and resilient urban environments.

### 3.1. Hot and Humid Cities: Urban Microclimate and Vegetation Analysis

This section summarizes the most recent advancements in urban microclimate studies (Table 1), highlighting cutting-edge methodologies and findings in vegetation coverage and thermal comfort analysis. These insights provide a foundation for contextualizing earlier studies and identifying emerging research trends. The analysis includes studies from 2017 to 2024, complementing recent advancements by offering foundational insights into vegetation’s role in urban microclimate dynamics. Together, these studies offer a comprehensive understanding of the topic’s evolution.

The impact of vegetation on urban microclimates has been widely studied, with a particular focus on mitigating the urban heat island effect [10]. Numerous studies have established the influence of urban vegetation on microclimate conditions [11]. Vegetation plays a critical role in reducing surface temperatures and enhancing thermal comfort, acting as a natural shading element, lowering ambient air temperatures through evapotranspiration, and improving air quality by filtering pollutants [12]. Vegetation in urban areas significantly influences the microclimate [13]. Multiple studies have examined the correlation between urban vegetation and meteorological parameters, including temperature, humidity, and wind speed [13,14,15]. Urban vegetation can improve humidity, lower wind speed, and cool the atmosphere. However, a reduction in urban vegetation coverage has been noted due to rapid urbanization [16]. The form and layout of urban vegetation, including its size, quantity, and spatial arrangement, affect its cooling potential. Overall, urban vegetation holds promise for mitigating the adverse effects of climate change [17].

**Table 1:** Recent advances in urban microclimate studies and vegetation analysis (2023–2024).

Ref	Methodology	Study Objectives	Main Findings	Gaps and Recommendations
[18]	- Employed Landsat satellite imagery from the USGS Surface Reflectance Climate Data Records to analyze land use and land cover variations. - Used supervised classification techniques for categorizing land use and cover types. - Computed land surface temperatures (LSTs) using a mono-window algorithm applied to the Landsat thermal bands. - Analyzed correlations between land use and LST using indices like NDVI, NDBI, NDWI, and NDBal.	- Investigate how spatiotemporal changes in land use and cover influence land surface temperatures. - Provide insights into urban climate resilience strategies. - Serve as a pioneering study in Qatar, a rapidly urbanizing Gulf nation.	- Built-up areas expanded by 343.16% from 2000 to 2023, primarily replacing deserts and water bodies. - Summer LST increased by 7.64 °C (0.34 °C annually), while winter LST declined by 4.87 °C (0.22 °C annually). - Urban and desert areas showed consistently high LST values during summer and winter.	- Investigate extreme temperature fluctuations in Doha and propose mitigation strategies. - Assess the impact of temperature changes on human thermal comfort and livability. - Evaluate the role of urban planning, green infrastructure, and advanced cooling technologies in addressing LST issues. - Analyze the socio-economic effects of rising temperatures in Doha.
[19]	- Used the Centers for Disease Control and Prevention (CDC)’s Social Vulnerability Index (SVI) to identify socially vulnerable census tracts in Philadelphia. - Modeled urban characteristics using Local Climate Zone (LCZ) classification and 3D urban models. - Simulated outdoor thermal comfort in high- and low-vulnerability areas using the ENVI-met microclimate model, focusing on the Predicted Mean Vote (PMV) index.	- Explore the link between outdoor thermal comfort and urban morphology in socioeconomically diverse areas. - Create a GIS-based methodology integrating LCZ and ENVI-met tools. - Provide a straightforward method for addressing inequalities in thermal comfort and urban design.	- Increased tree and grass coverage reduced air temperatures by up to 1.5 °C and mean radiant temperatures by 31 °C during extreme heat events. - Urban morphology factors, including sky view factor (SVF) and height-to-width ratios, significantly influenced thermal comfort. - Areas lacking vegetation experienced higher radiant temperatures and discomfort.	- Extend simulation durations beyond 24 h for comprehensive microclimate analysis. - Enhance urban feature data for improved simulation accuracy. - Investigate indoor thermal comfort alongside outdoor conditions. - Conduct similar studies in diverse cities and climate zones to generalize findings.
[20]	- Collected environmental data (e.g., air temperature, humidity) during traverse campaigns in Lisbon using a mobile weather station. - Focused measurements on summer afternoons across six study areas. - Assessed Universal Thermal Climate Index (UTCI) patterns and conducted hotspot analysis using GIS-based Getis-Ord Gi * statistics and 3D mapping.	- Examine UTCI behavior under various urban density and shading conditions. - Identify and map UTCI hotspots in Lisbon using GIS methods.	- Eleven UTCI hotspots were found in moderate to high-density urban areas, often in busy streets or open spaces with materials like tar and concrete. - Higher UTCI values were consistently observed in denser areas compared to low-density zones.	- Address limitations in the LCZ approach, including its inability to account for topography and wind patterns. - Conduct more in-depth studies on urban morphology’s effects on thermal comfort using both objective and subjective methods.
[21]	- Designed a street-level indicator system to describe urban morphology, covering aspects like land use, building geometry, and ecological landscape distribution. - Quantified factors using GIS, satellite imagery, and spatial data for Shanghai.	- Develop methods to improve urban microclimates and air quality. - Identify key urban morphology factors affecting PM2.5 dispersion and urban ventilation. - Propose optimization strategies for urban planning and design.	- Identified eight dominant factors influencing urban ventilation and air quality, including vegetation cover, spatial congestion, and building density. - Recommended source control, diversion, and convergence strategies to mitigate urban morphological impacts on pollution.	- Introduce additional parameters such as air temperature, wind direction, and humidity to integrate urban spatial planning with ecological systems. - Explore how enhanced urban planning can improve microclimates and living conditions.

#### 3.1.1. Urban Tree Canopy Cover, Temperature, and Humidity Levels Correlations

Understanding the correlations between urban tree canopy cover, temperature, and humidity levels is essential for urban planning and environmental management [22]. Research has indicated that increasing tree canopy coverage within urban areas effectively mitigates the urban heat island phenomenon by providing shade and cooling the surrounding environment [23]. A notable correlation exists between urban tree canopy cover and temperature and humidity levels [24]. Studies have demonstrated that urban areas with more extensive tree canopy cover experience lower temperatures and higher humidity levels than regions with less vegetation [25]. This phenomenon is attributed to trees providing shade, which reduces the heat absorption of buildings and pavement, thereby lowering temperatures in urban areas.

- Temperature: Trees influence the microclimate through factors such as air temperature and solar radiation, regulating leaf surface temperature [26]. Street trees with a high leaf area index (LAI) and larger canopies offer more cooling benefits during the day [27]. A higher percentage of canopy cover lowers daily temperatures, particularly in rural areas [28].
- Humidity: Relative humidity has less impact on leaf surface temperature (LST) compared to solar radiation and air temperature [26]. Under tree canopies, temperature drops are negatively correlated with vapor pressure deficit and wind speed [27]. The effect of leaf area index and canopy coverage may be outweighed by the impact of the vertical canopy structure, which significantly contributes to cooling impacts [29].

In summary, the extent of the tree canopy cover directly affects temperature and humidity levels. Trees influence microclimatic variables and provide cooling benefits.

#### 3.1.2. Sky View Factor Correlations of Street Canyons

**Figure 6:**
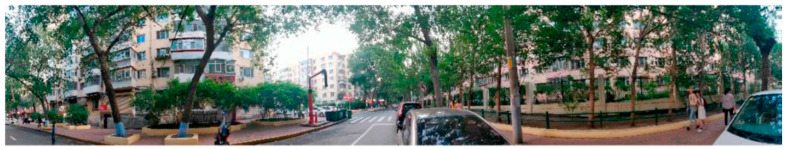
Street canyon: street view image (adapted from Ref. [30]).

A study conducted in Harbin, China, examined the relationship between the physical characteristics of the landscape and temperature conditions in street canyons. The Sky View Factor (SVF) was used as a key measure to regulate this correlation. The thermal environment was analyzed using numerical simulations to investigate the effects of various roadway canyon landscape topologies [30]. The findings demonstrated a statistically significant quadratic association between the street view factor and temperature, relative humidity, and mean radiant temperature (MRT) within the street canyon [31]. Moreover, the inclusion of street trees was found to be an efficient method for reducing urban heat and optimizing thermal conditions. The study also revealed a strong association between the morphology of street canyons, including their direction and aspect ratio, and outdoor thermal comfort [32]. As shown in Figure 6, the semi-dense street canyon in Harbin contributes to a cooler atmosphere due to its urban morphology and climatic conditions. This study used SVF as a key indicator to regulate street canyon morphology. Using the ENVI-met microclimate model, the researchers simulated various street canyon topologies and examined their effects on temperature, humidity, and mean radiant temperature. A significant quadratic relationship between SVF and temperature parameters was identified. Field measurements and regression analysis also revealed an inverse relationship between SVF and midnight ground surface temperature (GST) and a direct relationship between SVF and daytime GST across different street canyon configurations.

#### 3.1.3. GIS-Based Analysis in Studying Urban Microclimate

GIS-based analyses are increasingly utilized to study the impact of vegetation on urban microclimates [33]. Geographic Information Systems enable spatial analysis by integrating vegetation distribution data with microclimate information, helping to identify correlations and trends. GIS technology supports mapping, visualization, and spatial modeling, making it essential for understanding the complex relationship between vegetation and microclimate factors [34]. This GIS-based approach facilitates the evaluation of data sets, such as vegetation coverage, land use, and temperature, providing valuable insights into how vegetation influences the microclimate in urban areas.

Moreover, GIS can identify specific locations that are in need of intervention to alleviate the heat island effect and enhance environmental quality in urban areas [35]. A study using spatial analysis evaluated the influence of vegetation on the microclimate of a metropolitan area [36]. The researchers found that urban trees and plants significantly reduced the urban heat island effect by lowering surface temperatures [37]. Using GIS-based analysis, the researchers mapped and analyzed the spatial arrangement of vegetation within metropolitan regions, establishing connections with microclimate factors, including surface temperature, humidity, and wind dynamics [38]. GIS-based analysis has been used in numerous case studies to assess the influence of vegetation on urban microclimates. For example, research conducted in Singapore examined the spatial distribution of green areas and their impact on air temperature [39]. The findings showed that regions with more vegetation coverage had reduced air temperatures, demonstrating the cooling influence of green areas. Another study, using ArcGIS software, assessed the impact of vegetation on the urban microclimate in a specific city [40]. Urban trees and plants were found to significantly moderate the urban heat island effect by lowering land surface temperatures.

Several studies have employed GIS technology to assess various aspects of urban environments in Saudi Arabia. For example, a study in the Taif region used GIS and multiple-criteria decision analysis to evaluate the suitability of urban green spaces, identifying proximity to water, roads, precipitation, and land use as key factors [41]. The study found that 56.4% of the region was suitable for green space development, particularly in the southwestern areas. Another GIS-based study in the Al Ahsa Metropolitan Area analyzed land use and predicted settlement expansion, revealing an 18% decrease in vegetation due to urban growth [42]. These studies highlight GIS’s role in sustainable urban design and planning.

#### 3.1.4. Urban Heat Island Mitigation

Mitigating the urban heat island effect is essential for creating more sustainable and livable cities. Vegetation plays a pivotal role in reducing the urban heat island phenomenon, with evapotranspiration and shading helping to improve urban microclimates by facilitating cooling. Additionally, incorporating white roofs and suitable architectural designs can improve cooling efficacy and alleviate the consequences of urban heat islands [43]. Vegetation cools the environment through two primary mechanisms: evapotranspiration and shading. These processes reduce heat absorption by surfaces and increase water evaporation, thus lowering temperatures in urban areas [44,45]. Studies have demonstrated that increasing vegetation cover in urban areas can decrease surface and air temperatures, particularly during heatwaves [46]. Urban greening strategies, such as green roofs, vertical gardens, and urban forests, have been identified as effective approaches to mitigating the urban heat island effect and enhancing thermal comfort [47]. As urban heat islands and thermal discomfort increasingly affect urban environments, modeling approaches such as ENVI-met and agent-based modeling (ABM) have become essential tools for evaluating heat mitigation strategies. These models enable a detailed analysis of microclimatic changes and pedestrian behavior under various urban design interventions. The study emphasizes urban microclimates and seeks to offer insights into how specific techniques can enhance thermal conditions and comfort levels for pedestrians [48]. In this study, an integrated framework utilizing ENVI-met and ABM was used to analyze the effects of heat mitigation measures, such as cool pavements, shading structures, and green roofs, on outdoor microclimates, thermal comfort, and walkability. ENVI-met simulations accurately predicted air temperature but showed slight inconsistencies in wind speed and humidity due to urban complexity. The results demonstrated that cool pavements could reduce peak air temperatures by 0.36 °C, while street trees lowered the mean radiant temperature by 4.23 °C, emphasizing the importance of multifaceted strategies for effective urban heat mitigation. Additionally, the analysis revealed that the high Universal Thermal Climate Index (UTCI) values in the study area exposed pedestrians to heat stress, with street trees reducing the UTCI by 0.88 °C. This study employed a case study approach, focusing on a selected region in Hong Kong, to assess various heat mitigation strategies. It evaluated the effectiveness of urban greening, shade structures, and cool materials in reducing heat and improving the thermal environment. Data were collected through onsite measurements, computational models, and surveys on human perception. The findings underscored the critical need to integrate urban forms and vegetation in infrastructure planning to enhance thermal comfort and urban resilience [48].

#### 3.1.5. Esthetic Impact of Vegetation

In addition to mitigating urban heat, urban vegetation provides significant esthetic benefits. Green spaces and plants in metropolitan areas can improve esthetics, support mental health, and foster a sense of community [49,50]. Furthermore, green spaces serve as natural habitats for wildlife, playing a crucial role in preserving biodiversity within urban environments [51]. Studies have examined the role of vegetation in urban planning and its influence on the visual appeal of urban areas [52]. Research has demonstrated that incorporating vegetation into urban settings enhances esthetic perception.

Moreover, vegetation in metropolitan regions has been shown to improve air quality by reducing pollutants and purifying toxic compounds [53]. Green spaces offer various mental health benefits, including stress reduction and mood improvement, making them crucial components in developing livable and sustainable urban areas [54]. Research indicates that integrating plants into urban architecture yields numerous environmental advantages, such as decreasing air pollution, reducing the urban heat island effect, and creating habitats for wildlife. Edward O. Wilson’s theory of biophilia postulates that people have an innate connection to the natural world [55,56]. The presence of natural elements, such as vegetation, can significantly enhance esthetics and improve visual appeal. Thus, incorporating vegetation into urban planning not only improves visual esthetics but also fosters a healthier and more content urban population [57].

### 3.2. Cities in Saudi Arabia

#### 3.2.1. Humanization of the Streets: Tree Planting Initiative for Eastern Province Cities

The Tree Planting Initiative aims to address the environmental challenges faced by the cities of Khobar and Dammam. Through tree planting, this initiative seeks to improve air quality, reduce pollutants, and enhance the esthetic appeal of urban areas. Additionally, it encourages community engagement and motivates residents to actively participate in preserving the environment for future generations. The Khobar Municipality has launched the “A Tree for Every House” initiative, which offers trees to residents free of charge. This program aims to promote environmental awareness and enhance the city’s green spaces. By participating, residents can request a tree to be planted on their property, contributing to the development of a healthier and more sustainable community. One study highlighted the importance of green areas in reducing climate change impacts and improving human well-being, noting that green space availability per capita in Saudi cities is significantly below international standards [58].

**Figure 7:**
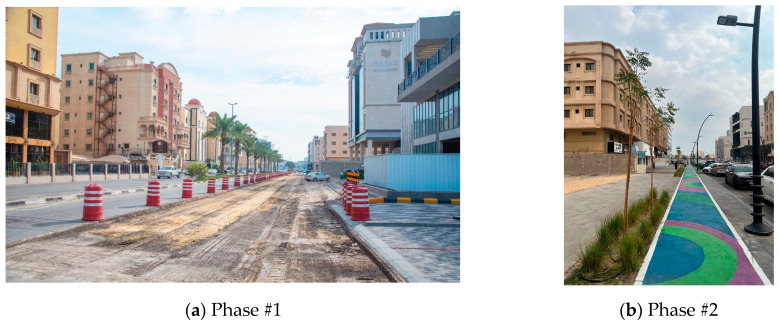
(**a**) Phase #1 Bashar Bin Burd Street, Olaya district (adapted from Ref. [61]); (**b**) Phase #2 21st Street, Olaya district (captured by the first author).

A GIS and spatial statistical analysis conducted in Abha and Bisha assessed the spatial distribution of environmental sustainability. The analysis, using standard distance measurements, revealed the clustering or dispersion of green spaces around the mean center, emphasizing the importance of green spaces in urban planning [59]. Another study found that physical activities for recreation or exercise on neighborhood streets are associated with perceived environmental qualities [60]. The development and humanization of Street 21 and Bashar Bin Burd in the Olaya district of Khobar city has begun (Figure 7). This project aims to enhance the quality of life in Khobar by integrating urban planning improvements focused on safety, accessibility, and esthetics. Key measures include standardizing traffic flow, constructing pedestrian sidewalks, planting trees along streets, and creating dedicated bicycle paths. These changes aim to encourage walking and cycling as healthier, more sustainable modes of transportation while improving road safety. Furthermore, the greenery and beautification efforts will enhance the city’s atmosphere, making it more inviting for residents and visitors and thus boosting Khobar’s appeal as a vibrant and sustainable urban environment [61].

**Figure 8:**
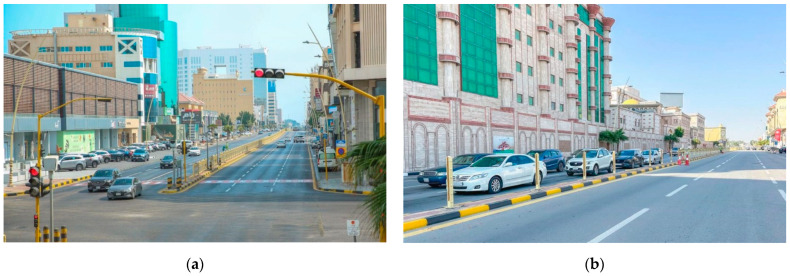
(**a**) Prince Faisal bin Fahd Road: Before removing the fences; (**b**) Prince Faisal bin Fahd Road (after removing the fences; adapted from Ref. [61]).

**Figure 9:**
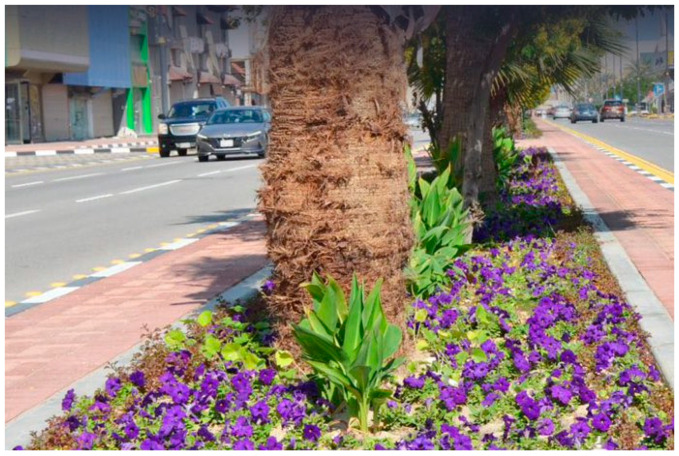
Eastern Province Municipality, Dammam City (adapted from Ref. [62]).

**Figure 10:**
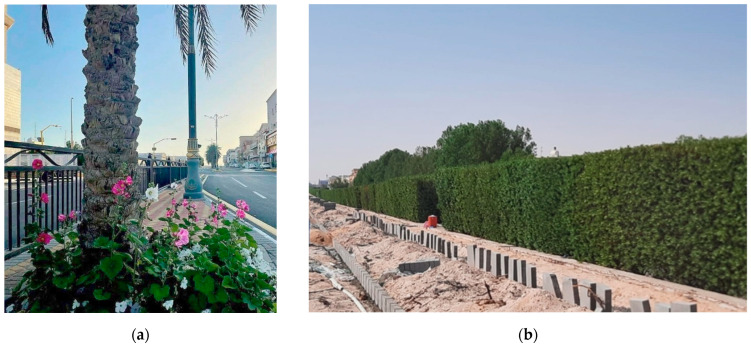
(**a**) Pedestrian track plantation at Abqaiq Governorate; (**b**) pedestrian track maintenance at Eastern Province Municipality, Abqaiq Governorate (adapted from Ref. [63]).

The development of Prince Faisal bin Fahd Road in Khobar is underway, involving tasks such as fence removal, center island enhancement, asphalt works, and planting over 450 trees along the road to improve the overall quality of life (Figure 8). The project includes the establishment of pedestrian sidewalks and a designated bicycle path to enhance accessibility and safety for residents. Additionally, the planting of trees along both sides of the road will improve visual appeal and contribute to a more environmentally sustainable atmosphere [61]. The Eastern Province Municipality is enhancing the esthetic appeal of Dammam by planting seasonal flowers in squares, highways, and central islands (Figure 9). The municipality is also responsible for maintaining and cleaning these planted areas to preserve their visual appeal. Efforts are underway to integrate sustainable landscaping techniques, including the use of indigenous flora that require less water and maintenance [62]. The Eastern Province Municipality recently conducted agricultural activities in the Abqaiq Governorate to increase vegetation cover, enhance the urban landscape, promote environmental sustainability, improve quality of life, and create more livable cities (Figure 10). The mayor of Abqaiq reported that 98,000 hybrid Petunia flowers and 30,000 Vinca rose plants were planted as winter and permanent annuals. Additionally, 868 trees and shrubs, including Neem, Ficus, Orzo, Bougainvillea, and Acacia, were planted [63].

#### 3.2.2. Vegetation Metrics and Microclimatic Modifications

We have explored various vegetation metrics to quantify their impact on microclimates, these include vegetation cover, green space density, and leaf area index [64,65]. Research has shown that higher vegetation cover and density correlate with reduced temperatures, increased humidity, and improved air quality [66]. Specific vegetation types, such as tree planting, have demonstrated significant cooling effects and energy savings [67]. A study conducted in Riyadh, Saudi Arabia, revealed that surface temperatures were reduced by approximately 4–5 °C within park areas compared to surrounding urban districts [68]. The presence of vegetation can enhance atmospheric humidity levels, as demonstrated by a GIS-based model simulating the influence of vegetation on metropolitan microclimates. The study found that a 10% increase in vegetation cover could decrease land surface temperature by 1–2 °C while increasing air humidity by 5–10% [69]. Previous studies also indicated that the benefits of vegetation were most pronounced in areas with high population density [70].

Due to climate change, urban valleys are generating urban heat islands. To improve outdoor thermal comfort, a study used an ENVI-met simulation to assess air temperature, wind speed, sky view factor, mean radiant temperature, and physiological equivalent temperature in an urban street [71]. The study also investigated the influence of tree species and street alignment. It found that the Sky View Factor does not precisely predict radiation or outdoor thermal comfort (OTC) symptoms. However, significant changes in orientation were found to dramatically improve OTC in urban valleys, highlighting the importance of appropriate solutions.

The study combined onsite observations, computer models, and statistical analyses to evaluate the impact of roadway orientation and tree species on thermal conditions. The research was conducted in a hot and humid climate, which presents unique challenges for heat stress and maintaining thermal comfort. The impact of street orientation was assessed by examining the angle at which roadways align with the sun’s path. Urban canyons experience varying levels of solar radiation exposure and shading patterns depending on their orientations. The study explored the correlation between street orientation and various thermal comfort indicators, including air and radiant temperatures.

By optimizing tree layout in roadway canyons, cooling effects can be maximized, and thermal comfort improved. The canopy diameter of trees should be maximized, and trees with leaf area densities greater than 1.5 m^2^/m^3^ should be selected [72]. Tree configuration in deeper street canyons may be less significant when nearby buildings provide shade, further enhancing outdoor thermal comfort [73]. Regarding outdoor thermal comfort, factors such as foliage size and shape, leaf area density, and seasonal variation are crucial [74]. Tree species exhibit varying impacts on temperature reduction and thermal comfort. For instance, coniferous trees planted along sidewalks have less impact, while deciduous trees in median strips can significantly enhance air quality and thermal comfort [75]. To optimize the microclimate and improve outdoor thermal comfort, the main street should follow the prevailing wind direction [73]. The aspect ratio of the roadway canyon and the spatial distribution of trees within it can also affect thermal comfort. Double-row tree planting patterns provide enhanced thermal comfort compared to center tree-planting patterns [76].

#### 3.2.3. Sustainable Development Goals Aligned with Saudi Vision 2030

Based on the insights from the previous case studies, it is clear that thorough research and analysis are essential for making informed decisions regarding heat mitigation in the local context, specifically Khobar City, Saudi Arabia. Before executing any plans, it is critical to examine the potential risks and implications. The successful implementation of the Urban Planning and Design Framework requires collaboration, data-driven decision-making, and a commitment to sustainable urban development. An examination of the role of GIS in Saudi Arabia, particularly in the context of Saudi Vision 2030, highlights its importance [77]. The analysis also investigates the SDGs that can be effectively tracked using GIS. Through GIS technology, Saudi Arabia can efficiently monitor and evaluate the progress of various SDGs outlined in the Saudi Vision 2030.

The SDGs provide a structured framework for evaluating the impact of vegetation on urban microclimates by focusing on specific objectives related to climate action, sustainable cities and communities, and life on land. These objectives can guide the process of data collection and geographical pattern analysis to understand vegetation’s impact on temperature, air quality, and overall quality of life in urban areas. The SDGs address factors such as vegetation diversity, urbanization, land restoration, forest monitoring, water-related ecosystems, sustainable urban development, land allocation, and coastal and rural planning [78,79,80,81,82,83]. These trends are crucial for achieving SDGs 11, 13, and 15, which focus on sustainable cities, climate action, and life on land. Prioritizing urbanization opportunities and land use planning is key to achieving these goals [84]. Furthermore, vegetation cover is critical to achieving these SDGs [80,85]. The relationship between changes in plant cover and urbanization, land use, and land cover patterns is strongly interconnected. This emphasizes the importance of vegetation in the context of sustainable development [86,87].

In the context of Saudi Arabian cities, various studies employ diverse methodologies focusing on green spaces, environmental challenges, and green innovation. However, significant gaps remain, particularly in qualitative assessments, comparative analyses, and the incorporation of public perceptions. Table 2 summarizes recent studies on urban green development in Saudi cities, identifying key themes and research gaps. These studies collectively emphasize the importance of green spaces for environmental sustainability, human well-being, and the achievement of Saudi Vision 2030. However, the differing approaches and contexts reveal nuanced insights and discrepancies.

Several studies agree that green spaces are crucial for reducing the impacts of climate change and enhancing urban sustainability. For example, References [58,88] both highlight the per capita green space deficiency in Saudi cities compared to international standards, advocating for innovative urban greening strategies such as vertical greening and advanced adaptation mechanisms. Similarly, studies like References [41,59] emphasize the importance of spatial planning and data integration in optimizing green space distribution, presenting green infrastructure as a key solution to both environmental and social challenges.

Despite these commonalities, some studies diverge in their focus and methodologies, leading to contrasting findings. For instance, Reference [41] identifies rainfall, proximity to roads, and water sources as primary suitability criteria for green space in Taif, while References [88,89] highlight the negative impacts of urban sprawl on green cover, and Reference [90] shifts the focus to the economic and social benefits of green entrepreneurship, offering different perspectives on sustainable development. These variations reflect how geographic and ecological differences shape green space strategies. Moreover, after synthesizing these finding, a multidimensional understanding of urban green development in Saudi cities can be obtained. The studies collectively stress the need to integrate green spaces into urban planning frameworks, recommending spatial–temporal mapping [58], longitudinal studies [59], and advanced modeling tools [88,93]. However, gaps persist, such as the underrepresentation of socio-economic and cultural factors [91], limited community engagement [92], and inadequate policy integration [41,90]. These gaps suggest opportunities for more holistic and inclusive approaches to urban green development. Future research can address these gaps by combining the spatial methodologies of [41,92] with stakeholder engagement strategies from [88,93]. Additionally, examining the broader socio-economic impacts, as proposed in [90,91], can align green initiatives with the broader goals of sustainable development in Saudi Arabia.

Saudi Arabian cities face a significant deficiency in per capita green space compared to international benchmarks. Table 3 summarizes studies on green spaces, environmental sustainability, and urban growth in the Kingdom, focusing on techniques, objectives, findings, and identified research gaps. Survey-based approaches to capture public perceptions, spatial analyses to map green space distribution, and the use of GIS and remote sensing for investigating land use changes are among the most prominent methodologies.

The research shares common goals of promoting urban sustainability, green innovation, and environmental resilience, particularly regarding climate change mitigation and well-being enhancement. The findings highlight the varied benefits of green spaces, such as their ability to improve thermal comfort, ecological health, and urban livability. They also demonstrate the positive impact of green innovation on economic, social, and environmental outcomes. Despite these advancements, significant gaps remain. Most studies focus primarily on quantitative assessments, neglecting qualitative insights or cross-city comparisons. Furthermore, inadequate stakeholder engagement and the limited use of new analytical tools, such as machine learning, constrain the broader applicability of the findings. To address these gaps, future research should employ mixed-method techniques, consider socio-cultural and ecological elements such as soil quality and biodiversity, and conduct longitudinal and multi-city assessments to better inform sustainable urban development plans.

The methodologies used across these studies reveal a distinct preference for GIS, remote sensing, and surveys/questionnaires. GIS and remote sensing excel at spatial analysis and mapping, capturing large-scale patterns and land use changes, while surveys provide valuable insights into public perceptions and behavioral trends. While these methodologies complement each other, they highlight a gap in integrating qualitative insights with quantitative data. Although many studies utilize multi-criteria decision analysis (AHP) or statistical models, fewer employ participatory methodologies to explore public perceptions or local cultural dynamics, indicating a need for more inclusive research approaches in the future.

Most studies share a common goal of promoting urban sustainability and enhancing environmental performance through green spaces. However, their specific objectives differ. For example:

- Climate Change Mitigation and Adaptation: Studies such as [58,88] focus on the role of green spaces in addressing climate challenges, emphasizing strategies for resilience and thermal comfort.
- Green Innovation and Urban Sustainability: Other works, including [90,94], examine the economic and social dimensions of green initiatives, particularly in small and medium enterprises (SMEs). This divergence highlights the need for interdisciplinary approaches that connect the environmental, economic, and social dimensions.

A strong consensus emerges on the critical role of green spaces in mitigating urban heat, enhancing well-being, and fostering sustainability. Other aspects that were noted as as follows:

- GIS-based studies [41,42,95] emphasize spatial distribution and suitability for green space development, often identifying environmental challenges such as urban sprawl and natural resource degradation.
- Survey-based studies [90] reveal positive public attitudes toward green initiatives but underscore barriers such as poor infrastructure and adverse climate conditions. Despite these shared findings, the scope of analysis varies, with some studies focusing on macro-level spatial trends [42,96], while others focus on micro-level behavioral patterns.

Several research gaps are identified across the studies:

1. Lack of Comparative and Temporal Analyses: Few studies compare green space strategies across cities or assess long-term changes [42,59]. Addressing this could provide benchmarks and identify best practices.
2. Limited Public and Stakeholder Engagement: While many studies acknowledge the importance of perceptions [41,58], they rarely integrate public participation into decision-making, missing critical cultural and local insights.
3. Inadequate Use of Advanced Tools: Recommendations for incorporating tools like machine learning and extending the criteria to include soil quality, biodiversity, and cultural factors [41,97] highlight the potential for more nuanced and accurate analyses.

The comparative analysis reveals broad agreement on the need for green spaces and innovative strategies for urban sustainability but underscores methodological and contextual gaps. Future research should incorporate the following:

- Integrate Quantitative and Qualitative Approaches: Combining GIS/spatial methods with participatory and mixed-method frameworks can provide a holistic understanding of green space dynamics.
- Expand Criteria for Evaluation: Incorporating socio-cultural and ecological factors alongside advanced analytical tools can enhance the depth and relevance of the findings.
- Encourage Comparative and Longitudinal Studies: Investigating multiple cities and temporal trends will enable the identification of scalable and adaptable strategies for Saudi cities.

#### 3.2.4. Recommendations for Enhancing Khobar’s Tree Planting Initiative

The findings of this study offer a comprehensive set of recommendations aimed at maximizing the effectiveness of the Tree Planting Initiative in Khobar City, Saudi Arabia, as outlined in Table 4. These proposals focus on improving urban microclimates and community participation through strategic vegetation management. They emphasize refining plant species selection, expanding educational outreach, and establishing effective maintenance and monitoring strategies. By integrating these components, the initiative aims to enhance the esthetics of the urban environment while strengthening the ecological and social fabric of the city. These actions will lay a robust foundation for sustainable urban development, ensuring long-lasting benefits for both the environment and the community.

The recommendations outlined in this study are designed to integrate strategic urban vegetation practices into the planning frameworks of Saudi cities, aligning with national objectives such as Saudi Vision 2030. These solutions encompass prioritizing indigenous and drought-resistant plants, establishing green corridors, and utilizing GIS-based monitoring tools to guarantee sustainable execution and efficient maintenance. The proposals underscore the significance of community involvement via educational programs, workshops, and participatory efforts to cultivate a sense of ownership and enduring dedication to greening projects. This recommendation seeks to promote policies that align with sustainability objectives and integrate global best practices suited to the environmental and socio-economic context of Saudi Arabia, with the intent to improve urban microclimates, alleviate the urban heat island effect, and advance the overarching aims of sustainable urban development.

## 4. Conclusions

This review emphasizes the critical role of urban vegetation in mitigating the UHI effect and improving microclimatic conditions, particularly in hot and humid urban environments. By analyzing case studies and synthesizing the global literature, the study highlights how vegetation contributes to thermal comfort, ecological resilience, and urban esthetics.

Key findings indicate that strategic urban greening initiatives—such as tree planting, optimizing street orientation, and integrating green corridors—are essential for reducing surface temperatures and creating livable cities. The review also identifies correlations between urban vegetation metrics, such as tree canopy cover and sky view factors, and their effectiveness in modifying microclimatic conditions. Furthermore, GIS-based tools and methodologies proved to be invaluable assets for spatial analysis and urban planning, enabling data-driven decision-making and supporting sustainable development. For urban planners and policymakers, this review provides actionable recommendations:

- Promote Native Vegetation: Prioritize drought- and heat-resistant species adapted to local climates to maximize ecological and thermal benefits while minimizing resource use.
- Enhance Community Engagement: Foster public participation in urban greening initiatives to align with local needs and encourage long-term stewardship.
- Leverage Technology: Employ advanced tools, such as GIS and remote sensing, to analyze and monitor urban vegetation and its microclimatic impacts.
- Align with SDGs: Integrate urban greening efforts with the SDGs, particularly Goal 11 (Sustainable Cities and Communities) and Goal 13 (Climate Action), to ensure broader environmental and societal benefits.

The findings emphasize the potential of urban greening to create more resilient, climate-adaptive cities. However, gaps remain, such as the need for longitudinal studies, stakeholder-driven strategies, and comparative analyses across various urban contexts.

In conclusion, urban vegetation offers a multifaceted approach to addressing the challenges of hot and humid urban climates. By adopting a holistic perspective that integrates ecological, technological, and social dimensions, urban planners can pave the way for more sustainable, resilient, and equitable urban environments. This synthesis serves as a foundation for future research and practical applications, fostering a synergy between nature-based solutions and sustainable urban development.

Future studies should focus on cross-city comparisons and longitudinal analyses to identify sustainable best practices for green infrastructure in hot climates. Integrating advanced analytical tools, such as GIS and machine learning, will enhance spatial studies and support data-driven urban planning. Building on the findings of this review, ongoing experimental research using ENVI-met is evaluating the microclimatic effects of vegetation in hot urban environments. These efforts aim to validate theoretical insights and refine strategies to optimize the impact of urban vegetation on sustainability and livability.

## Figures and Tables

**Table 2:** Current state of the literature on the urban green development of Saudi cities.

Ref.	Methodology	Study Objectives	Main Findings	Gaps and Recommendations
[58]	A semi-structured questionnaire with 18 questions on 6 green space strategies and per capita green space calculations.	Exploration and assessment strategies for green spaces to promote urban environmental sustainability in Saudi Arabian cities.	- Green spaces are crucial in mitigating climate change and improving human well-being. - A considerable shortfall in the per capita availability of green spaces in Saudi cities compared to international standards.	- Implementation of innovative urban greening strategies like green roofs and vertical greening. - Spatial–temporal mapping of green spaces to aid in landscape planning. - Incorporating urban greening strategies into the decision-making frameworks of local governments.
[59]	Analytical descriptive methodology and substantive, regional, and historical approaches, creating a geodatabase using ArcCatalog to enable spatial statistical analyses.	Investigate the importance of environmental sustainability and its contribution to Saudi Vision 2030 in two cities, Abha and Bisha, to identify the contributing parameters.	- Recognition of the importance of green spaces is essential for policymakers in urban planning and development. - The standard distance measurement indicated the clustering or dispersion of green spaces around the mean center.	- Conducting longitudinal studies to track changes in green areas and environmental sustainability in Abha and Bisha over time. - Comparing the findings from Abha and Bisha with those of other cities in Saudi Arabia or globally to identify best practices for promoting environmental sustainability through green areas.
[41]	Integrating various spatial datasets, e.g., climatic, morphological, topographic, and land use/land change, for developing multi-criteria decision-making.	Assessment of the suitability of urban green spaces in the Taif region of Saudi Arabia for the preservation of the natural environment	- The key criteria influencing the suitability of urban green spaces in the Taif region were distance to water, road distance, and rainfall. - The region demonstrated fair suitability for green space development across 56.4% of its total area, with higher percentages in the southwestern part.	- Lack of effective guidance for decision-makers on sustainable development of human and natural resources - Soil quality, biodiversity, cultural significance, and community preferences can be incorporated for a more holistic evaluation.
[88]	Online and field surveys with questionnaires and semi-structured interviews with four main sections (demographics, preferences for green space services, and their importance in climate change mitigation and adaptation).	- Assessment of the per capita availability of green spaces in Jeddah, Saudi Arabia. - Investigating green spaces in climate change mitigation and adaptation strategies.	- Most respondents strongly agreed with adaptation strategies related to climate change, particularly those that improve the per capita availability and accessibility of green spaces across cities. - There were substantial shortfalls in the per capita availability of GS in Jeddah compared to global standards.	- Use advanced machine learning tools and techniques to study patterns of green spaces. - Assess the role of green spaces during the summer and winter seasons to improve understanding.
[89]	Utilization of various remote sensing and GIS software and tools, including ERDAS IMAGINE, ENVI, ArcGIS, and QGIS, to process and analyze the remote sensing data.	Assessment of the environmental performance and impacts related to the changes in land use.	- Significant growth in agricultural land from 1975 to 2005, followed by a decline between 2005 and 2019, an increase in urban land over the years, and a decrease in reclaimed land from 1975 to 2019. - Highlighted environmental risks associated with urban sprawl and agricultural activities, including soil pollution, water pollution, agricultural pollution, valley contamination, flooding, and biodiversity loss.	- Limited focus on specific environmental impacts (air quality, habitat loss, and degradation of ecosystem services) due to agricultural activities. - Lack of socio-economic analysis due to land use changes, such as population dynamics, income levels, and land tenure systems. - Shortcomings in data accuracy and validation that could affect the reliability of the study’s findings. - Community engagement and stakeholder involvement are needed to enhance the relevance and applicability of the findings.
[90]	Utilize a survey questionnaire to understand perceptions and willingness to walk among the residents and conduct field visits to assess walking conditions in the neighborhoods.	- Examination of the distribution patterns of community services in the neighborhoods of Dhahran city - Assessment of the design and conditions of streets and sidewalks in the two neighborhoods.	- Neighborhood residents show a positive attitude towards walking, with a significant portion walking to facilities for recreation and health benefits. - Poorly designed sidewalks and hot weather during summer hinder some residents from walking regularly.	- Investigating the distance and impacts of various facilities on the walking behavior of residents in the two neighborhoods. - Examining the physical conditions of the residents and their willingness to walk. - Analyzing the conditions of the streets in other parts of the Dammam metropolitan area and other cities.
[91]	A survey questionnaire was used to collect data from the top managers of Saudi Arabia’s manufacturing industries.	- Investigation of the effects of internal and external environmental challenges on green innovations. - Considering the role of top management in providing evidence.	External pressures and internal driving forces positively affect green initiatives and other indices.	- Conduct longitudinal studies to examine how small and medium enterprises’ choice of green innovation strategy varies over time and across different stages of development. - Inclusion of stakeholders beyond top managers, such as accountants and regular employees. - Incorporate additional factors like environmental corporate social responsibility and human resources practices into the conceptual framework.
[92]	Utilization of the fixed effects panel data models to examine the influence of green and nongreen entrepreneurship on the economic, environmental, and social dimensions of sustainable development in 13 cities in Saudi Arabia.	- Exploration of the influence of green entrepreneurial activity on sustainable development. - Analyzing the role of entrepreneurship policy in the context of Saudi Arabia.	Green entrepreneurship positively contributes to the economic, social, and environmental components of sustainable development in Saudi Arabia.	- Research on the influence of formal institutions on green entrepreneurship needs to be founded on theory. - Need for more cross-sectional and longer-term analyses, investigating evidence from other developing countries in the GCC region, and extending the study’s time frame. - Use different proxies for social, environmental, and economic aspects to test the relationship between green entrepreneurship and sustainable development.
[93]	Utilization of satellite imagery from 1985 to 2017 to identify various land cover classes: vegetation, urban, land, dunes, and water bodies through image differencing strategies over time.	Depiction of the spatial variations in the oasis’s green cover over the past 32 years using two scenarios (quantification of agricultural area and vegetation change).	- Significant urban sprawl occurred within the old oasis, with 3200 hectares of bare lands being converted to urban areas during the initial stage. - Urban development rapidly expanded within the oasis’s vegetation region, occupying 1270 hectares by 2017.	- The economic impact of vegetation degradation was not studied due to a lack of relevant data. - Changes in hydrogeological conditions and their effect on the oasis ecosystem and microclimate were not included as factors influencing the oasis’s demography.

**Table 3:** Comparative analysis of current studies: key elements.

Methodologies	Objectives	Main Findings	Gaps and Recommendations
Surveys and Questionnaires: Commonly used to gather data on perceptions, preferences, and attitudes [58,88,90,91,94]	Spatial Analysis and Mapping: Many studies aim to analyze spatial heterogeneity and distribution patterns of green spaces and environmental features [41,58,59,95,96]	Significant Role of Green Spaces: Green spaces are crucial for mitigating climate change, enhancing well-being, and supporting urban sustainability [41,42,58]	Quantitative vs. Qualitative Assessments: Many studies have focused on quantitative assessments; there is a need for qualitative evaluations and strategies for provisioning green spaces [58]
GIS and Remote Sensing: Widely utilized for spatial analysis, mapping, and land use/land cover changes [41,42,59,89,95]	Urban Sustainability and Green Innovation: Focus on promoting urban environmental sustainability and assessing the role of green innovation in SMEs [91,92,94]	Environmental Challenges and Recommendations: Various studies highlight environmental challenges such as urban sprawl, the degradation of natural resources, and the need for sustainable urban planning [42,95,97]	Comparative Studies Across Cities: A need for studies comparing green spaces and sustainability strategies across different cities rather than focusing on single-city analyses [58]
Multi-Criteria Decision Analysis (AHP): Employed for evaluating suitability and making informed decisions [41,96]	Climate Change and Environmental Impacts: Address the role of green spaces in climate change mitigation and adaptation [58,88]	Positive Impact of Green Innovation: Green innovation positively influences economic, social, and environmental performance, particularly in SMEs [91,94]	Perceived Statements and Public Participation: Incorporating public perceptions and stakeholder participation in future research can provide valuable insights [41,58]
Statistical Analyses: Applied to assess relationships, influences, and trends [91,92,94]	Comparative and Temporal Analysis: Some studies conduct comparative analyses between different cities or examine changes over time [42,59]	Longitudinal and Mixed-Method Studies: Longitudinal studies and mixed-method approaches can offer deeper insights into dynamic changes and validate findings [91,92,94]	Extended Criteria and Advanced Tools: Future research should consider additional factors like soil quality and cultural significance and use advanced tools like machine learning for comprehensive analysis [41,88,96]

**Table 4:** Comprehensive framework for urban greening and microclimate optimization.

Key Areas	Recommended Actions
Selection of plant species	Prioritize native and drought-resistant species to match local climatic conditions (context analysis).
	Conduct soil assessments to determine optimal planting conditions (data collection and site-specific studies).
	Perform microclimate impact studies to evaluate vegetation’s effect on thermal comfort (utilize technology).
Community engagement and education	Organize workshops and training sessions to educate residents and stakeholders (promote sustainable development and community participation).
	Collaborating with local schools and businesses, and environmental experts to foster community participation (stakeholder engagement).
	Schedule community tree-planting events to build awareness and commitment (community engagement and participation).
Maintenance and monitoring	Develop a GIS-based tracking system to monitor tree growth and health (utilize technology).
	Conduct regular maintenance checks to ensure long-term success (policy integration and regular monitoring).
	Establish a feedback mechanism for community input and continuous improvement (promote sustainable development).
Strategic planning and implementation	Integrate findings from the global literature and best practices into planning processes (literature review).
	Design and implement green corridors and urban green spaces for better connectivity and climate adaptation (integrate green infrastructure and heat-reduction techniques).
	Foster public–private partnerships to ensure sufficient resources and broad support (stakeholder engagement).
Policy and governance	Advocate for urban greening policies to institutionalize sustainable practices (policy integration).
	Ensure long-term government commitment to urban greening initiatives (policy integration).
	Engage with environmental agencies for research collaboration and support (stakeholder engagement).
Climate adaptation and resilience	Implement climate-adaptive strategies to mitigate urban heat and promote resilience (identify key issues and develop design principles).
	Foster research collaborations with academic and governmental entities for innovative solutions (foster research collaborations).
	Promote sustainable landscaping practices to conserve resources and enhance urban biodiversity (adaptability).

## Data Availability

Data will be made available upon request.

## References

[1] Dissanayake C., Weerasinghe U., Wijesundara K. Urban Vegetation and Morphology Parameters Affecting Microclimate and Outdoor Thermal Comfort in Warm Humid Cities—A Review of Research in the Past Decade. Proceedings of the 5th International Conference on Climate Change.

[2] (2023). Al-Riyadh Newspaper “Dar & Emaar” Contributes to Supporting the “Green Riyadh” Initiative.

[3] Al-Riyadh Newspaper 20 Million Riyals Were Allocated to Plant Trees in the “Saraya al-Narjis” Community. https://darwaemaar.com/.

[4] Olanrewaju R. (2009). The Climate Effect of Urbanization in A City of Developing Country: The Case Study Of Ilorin, Kwara State, Nigeria. Ethiop. J. Environ. Stud. Manag..

[5] Han D., Zhang T., Qin Y., Tan Y., Liu J. (2023). A Comparative Review on the Mitigation Strategies of Urban Heat Island (UHI): A Pathway for Sustainable Urban Development. Clim. Dev..

[6] Shao H., Kim G. (2022). A Comprehensive Review of Different Types of Green Infrastructure to Mitigate Urban Heat Islands: Progress, Functions, and Benefits. Land.

[7] Wai C.Y., Tariq M.A.U.R., Muttil N. (2022). A Systematic Review on the Existing Research, Practices, and Prospects Regarding Urban Green Infrastructure for Thermal Comfort in a High-Density Urban Context. Water.

[8] Wang X., Zhou Z., Xiang Y., Peng C., Peng C. (2024). Effects of Street Plants on Atmospheric Particulate Dispersion in Urban Streets: A Review. Environ. Rev..

[9] Page M.J., McKenzie J.E., Bossuyt P.M., Boutron I., Hoffmann T.C., Mulrow C.D., Shamseer L., Tetzlaff J.M., Akl E.A., Brennan S.E. (2021). The PRISMA 2020 Statement: An Updated Guideline for Reporting Systematic Reviews. BMJ.

[10] Chen Y., Zheng B., Hu Y. (2020). Mapping Local Climate Zones Using ArcGIS-Based Method and Exploring Land Surface Temperature Characteristics in Chenzhou, China. Sustainability.

[11] Duarte D.H.S., Shinzato P., dos Gusson C.S., Alves C.A. (2015). The Impact of Vegetation on Urban Microclimate to Counterbalance Built Density in a Subtropical Changing Climate. Urban Clim..

[12] Gatto E., Ippolito F., Rispoli G., Carlo O.S., Santiago J.L., Aarrevaara E., Emmanuel R., Buccolieri R. (2021). Analysis of Urban Greening Scenarios for Improving Outdoor Thermal Comfort in Neighbourhoods of Lecce (Southern Italy). Climate.

[13] Dang C., Shao Z., Huang X., Cheng G., Qian J. (2023). Different Urbanization Levels Lead to Divergent Responses of Spring Phenology. Photogramm. Eng. Remote Sens..

[14] D’Isidoro M., Mircea M., Borge R., Finardi S., de la Paz D., Briganti G., Russo F., Cremona G., Villani M.G., Adani M. (2023). The Role of Vegetation on Urban Atmosphere of Three European Cities—Part 1: Evaluation of Vegetation Impact on Meteorological Conditions. Forests.

[15] He Q., Reith A. (2023). A Study on the Impact of Green Infrastructure on Microclimate and Thermal Comfort. Pollack Period..

[16] Zhang P., Dong Y., Ren Z., Wang G., Guo Y., Wang C., Ma Z. (2023). Rapid Urbanization and Meteorological Changes Are Reshaping the Urban Vegetation Pattern in Urban Core Area: A National 315-City Study in China. Sci. Total Environ..

[17] Fu J., Dupre K., Tavares S., King D., Banhalmi-Zakar Z. (2022). Optimized Greenery Configuration to Mitigate Urban Heat: A Decade Systematic Review. Front. Archit. Res..

[18] Patel S., Indraganti M., Jawarneh R.N. (2024). Land Surface Temperature Responses to Land Use Dynamics in Urban Areas of Doha, Qatar. Sustain. Cities Soc..

[19] Hashemi F., Poerschke U., Iulo L.D., Chi G. (2023). Urban Microclimate, Outdoor Thermal Comfort, and Socio-Economic Mapping: A Case Study of Philadelphia, PA. Buildings.

[20] Silva T., Lopes A., Vasconcelos J. (2024). A Micro-Scale Look into Pedestrian Thermophysiological Comfort in an Urban Environment. Bull. Atmos. Sci. Technol..

[21] Wang Y., Dai X., Gong D., Zhou L., Zhang H., Ma W. (2024). Correlations between Urban Morphological Indicators and PM2.5 Pollution at Street-Level: Implications on Urban Spatial Optimization. Atmosphere.

[22] Li Y., Lin D., Zhang Y., Song Z., Sha X., Zhou S., Chen C., Yu Z. (2023). Quantifying Tree Canopy Coverage Threshold of Typical Residential Quarters Considering Human Thermal Comfort and Heat Dynamics under Extreme Heat. Build Environ..

[23] Wang Y., Ni Z., Hu M., Chen S., Xia B. (2021). A Practical Approach of Urban Green Infrastructure Planning to Mitigate Urban Overheating: A Case Study of Guangzhou. J. Clean Prod..

[24] Kayet N., Pathak K., Chakrabarty A., Sahoo S. (2016). Spatial Impact of Land Use/Land Cover Change on Surface Temperature Distribution in Saranda Forest, Jharkhand. Model Earth Syst. Environ..

[25] Lanza K., Alcazar M., Durand C.P., Salvo D., Villa U., Kohl H.W. (2023). Heat-Resilient Schoolyards: Relations Between Temperature, Shade, and Physical Activity of Children During Recess. J. Phys. Act. Health.

[26] Li R., Zeng F., Zhao Y., Wu Y., Niu J., (Leon) Wang L., Gao N., Zhou H., Shi X., Huang Z. (2023). Analyzing the Impact of Various Factors on Leaf Surface Temperature Based on a New Tree-Scale Canopy Energy Balance Model. Sustain. Cities Soc..

[27] Sharmin M., Tjoelker M.G., Pfautsch S., Esperón-Rodriguez M., Rymer P.D., Power S.A. (2023). Tree Traits and Microclimatic Conditions Determine Cooling Benefits of Urban Trees. Atmosphere.

[28] Vasconcelos V.V., Sacht H.M. (2020). Influence of Canopy Cover on Surface Temperature. Rev. Bras. Geogr. Fis..

[29] Wang X., Rahman M.A., Mokroš M., Rötzer T., Pattnaik N., Pang Y., Zhang Y., Da L., Song K. (2023). The Influence of Vertical Canopy Structure on the Cooling and Humidifying Urban Microclimate during Hot Summer Days. Landsc. Urban Plan..

[30] Li G., Ren Z., Zhan C. (2020). Sky View Factor-Based Correlation of Landscape Morphology and the Thermal Environment of Street Canyons: A Case Study of Harbin, China. Build. Environ..

[31] Li G., Cheng Q., Zhan C., Yocom K.P. (2022). Evaluation Strategies on the Thermal Environmental Effectiveness of Street Canyon Clusters: A Case Study of Harbin, China. Sustainability.

[32] Ghaffour W., Ouissi M.N., Velay Dabat M.A. (2020). Analysis of Urban Thermal Environments Based on the Perception and Simulation of the Microclimate in the Historic City of Tlemcen. Smart Sustain. Built Environ..

[33] Revollo N.V., Noelia Revollo Sarmiento G., Andrea Huamantinco Cisneros M., Delrieux C.A., Piccolo M.C. (2019). Assessing the Evolution in Remotely Sensed Vegetation Index Using Image Processing Techniques. Anu. Inst. Geocienc..

[34] El-Sheikh M.A., Thomas J., Alfarhan A.H., Alatar A.A., Mayandy S., Hennekens S.M., Schaminėe J.H.J., Mucina L., Alansari A.M. (2017). SaudiVeg Ecoinformatics: Aims, Current Status and Perspectives. Saudi J. Biol. Sci..

[35] Linhares C.S.F., Gonçalves R., Martins L.M., Knapic S. (2021). Structural Stability of Urban Trees Using Visual and Instrumental Techniques: A Review. Forests.

[36] Buyadi S.N.A., Mohd W.M.N.W., Misni A. (2013). Green Spaces Growth Impact on the Urban Microclimate. Procedia Soc. Behav. Sci..

[37] Ibrahim M., Koch B., Datta P. (2021). Evaluate the Effect of Land Surface Temperature in Arid and Semi-Arid Lands Using Potential Remote Sensing Data and GIS Techniques. Int. J. Glob. Warm..

[38] Liu X., Huang B., Li R., Zhang J., Gou Q., Zhou T., Huang Z. (2022). Wind Environment Assessment and Planning of Urban Natural Ventilation Corridors Using GIS: Shenzhen as a Case Study. Urban Clim..

[39] Teo Y.H., Makani M.A.B.H., Wang W., Liu L., Yap J.H., Cheong K.H. (2022). Urban Heat Island Mitigation: GIS-Based Analysis for a Tropical City Singapore. Int. J. Environ. Res. Public Health.

[40] Buyadi S.N.A., Mohd W.M.N.W., Misni A. (2014). The Effects of Vegetation Growth on the Microclimate of Urban Area: A Case Study of Petaling District. Geoinformation for Informed Decisions.

[41] Waheeb S.A., Zerouali B., Elbeltagi A., Alwetaishi M., Wong Y.J., Bailek N., AlSaggaf A.A., Abd Elrahman S.I.M., Santos C.A.G., Majrashi A.A. (2023). Enhancing Sustainable Urban Planning through GIS and Multiple-Criteria Decision Analysis: A Case Study of Green Space Infrastructure in Taif Province, Saudi Arabia. Water.

[42] Alqahtany A. (2023). GIS-Based Assessment of Land Use for Predicting Increase in Settlements in Al Ahsa Metropolitan Area, Saudi Arabia for the Year 2032. Alex. Eng. J..

[43] Zhang L., Yang Y., Lin Y., Chen H. (2022). Human Health, Environmental Quality and Governance Quality: Novel Findings and Implications From Human Health Perspective. Front. Public Health.

[44] Irfeey A.M.M., Chau H.W., Sumaiya M.M.F., Wai C.Y., Muttil N., Jamei E. (2023). Sustainable Mitigation Strategies for Urban Heat Island Effects in Urban Areas. Sustainability.

[45] Gunawardena K.R., Wells M.J., Kershaw T. (2017). Utilising Green and Bluespace to Mitigate Urban Heat Island Intensity. Sci. Total Environ..

[46] Alavipanah S., Wegmann M., Qureshi S., Weng Q., Koellner T. (2015). The Role of Vegetation in Mitigating Urban Land Surface Temperatures: A Case Study of Munich, Germany during the Warm Season. Sustainability.

[47] Arghavani S., Malakooti H., Ali Akbari Bidokhti A.A. (2020). Numerical Assessment of the Urban Green Space Scenarios on Urban Heat Island and Thermal Comfort Level in Tehran Metropolis. J. Clean Prod..

[48] Jia S., Wang Y. (2021). Effect of Heat Mitigation Strategies on Thermal Environment, Thermal Comfort, and Walkability: A Case Study in Hong Kong. Build. Environ..

[49] Semeraro T., Scarano A., Buccolieri R., Santino A., Aarrevaara E. (2021). Planning of Urban Green Spaces: An Ecological Perspective on Human Benefits. Land.

[50] Paudel S., States S.L. (2023). Urban Green Spaces and Sustainability: Exploring the Ecosystem Services and Disservices of Grassy Lawns versus Floral Meadows. Urban For. Urban Green..

[51] Threlfall C.G., Mata L., Mackie J.A., Hahs A.K., Stork N.E., Williams N.S.G., Livesley S.J. (2017). Increasing Biodiversity in Urban Green Spaces through Simple Vegetation Interventions. J. Appl. Ecol..

[52] Thorpert P., Rayner J., Haaland C., Englund J.E., Fransson A.M. (2022). Exploring the Integration Between Colour Theory and Biodiversity Values in the Design of Living Walls. Front. Ecol. Evol..

[53] Bikis A. (2023). Urban Air Pollution and Greenness in Relation to Public Health. J. Environ. Public Health.

[54] Barton J., Rogerson M. (2017). The Importance of Greenspace for Mental Health. BJPsych Int..

[55] Wilson E.O. (1984). Biophilia.

[56] Wilson E.O. (2019). Biophilia.

[57] Griffin D.R. (1985). Biophilia.

[58] Addas A., Maghrabi A. (2021). Role of Urban Greening Strategies for Environmental Sustainability—A Review and Assessment in the Context of Saudi Arabian Megacities. Sustainability.

[59] Abuhasel K. (2023). Geographical Information System Based Spatial and Statistical Analysis of the Green Areas in the Cities of Abha and Bisha for Environmental Sustainability. ISPRS Int. J. Geoinf..

[60] Aljohani N.M., Jaafar M., Choy L.K. (2021). Urban Planning in Al-Madinah Al-Munawarah Using New Green Spaces Modelling through GIS Application. Indones. J. Geogr..

[61] Khobar Municipality Khobar Municipality Streets Developments. https://www.eamana.gov.sa/.

[62] Eastern Province Municipality The Eastern Region is Decorated with Flowers and Trees Amidst Municipal Services Provided by the Eastern Province to Raise the Quality of Life. https://www.eamana.gov.sa/MediaCenter/Press/Pages/Magazine.aspx.

[63] Eastern Province Municipality Eastern Province Municipality Is Carrying Out a Number of Planting Works with Various Trees and Shrubs to Increase the Vegetation Cover in Abqaiq. https://www.eamana.gov.sa/MediaCenter/Press/Pages/Magazine.aspx.

[64] Sodoudi S., Zhang H., Chi X., Müller F., Li H. (2018). The Influence of Spatial Configuration of Green Areas on Microclimate and Thermal Comfort. Urban For. Urban Green..

[65] Rui L., Buccolieri R., Gao Z., Ding W., Shen J. (2018). The Impact of Green Space Layouts on Microclimate and Air Quality in Residential Districts of Nanjing, China. Forests.

[66] Zhu C., Ji P., Li S. (2017). Effects of Urban Green Belts on the Air Temperature, Humidity and Air Quality. J. Environ. Eng. Landsc. Manag..

[67] Kim H., Oh K., Lee D. (2021). Establishment of a Geographic Information System-based Algorithm to Analyze Suitable Locations for Green Roofs and Roadside Trees. Appl. Sci..

[68] Aina Y.A., Adam E.M., Ahmed F. (2017). Spatiotemporal Variations in the Impacts of Urban Land Use Types on Urban Heat Island Effects: The Case of Riyadh, Saudi Arabia. Int. Arch. Photogramm. Remote Sens. Spat. Inf. Sci.-ISPRS Arch..

[69] Alghamdi A.S., Alzhrani A.I., Alanazi H.H. (2021). Local Climate Zones and Thermal Characteristics in Riyadh City, Saudi Arabia. Remote Sens..

[70] Chen L., Ng E., An X., Ren C., Lee M., Wang U., He Z. (2012). Sky View Factor Analysis of Street Canyons and Its Implications for Daytime Intra-Urban Air Temperature Differentials in High-Rise, High-Density Urban Areas of Hong Kong: A GIS-Based Simulation Approach. Int. J. Climatol..

[71] Narimani N., Karimi A., Brown R.D. (2022). Effects of Street Orientation and Tree Species Thermal Comfort within Urban Canyons in a Hot, Dry Climate. Ecol. Inform..

[72] Yan S., Zhang T., Wu Y., Lv C., Qi F., Chen Y., Wu X., Shen Y. (2023). Cooling Effect of Trees with Different Attributes and Layouts on the Surface Heat Island of Urban Street Canyons in Summer. Atmosphere.

[73] Wu J., Chang H., Yoon S. (2022). Numerical Study on Microclimate and Outdoor Thermal Comfort of Street Canyon Typology in Extremely Hot Weather—A Case Study of Busan, South Korea. Atmosphere.

[74] Perini K., Chokhachian A., Auer T. (2018). Green Streets to Enhance Outdoor Comfort. Nature Based Strategies for Urban and Building Sustainability.

[75] Baradaran Motie M., Yeganeh M., Bemanian M. (2023). Assessment of Greenery in Urban Canyons to Enhance Thermal Comfort & Air Quality in an Integrated Seasonal Model. Appl. Geogr..

[76] Morakinyo T.E., Lam Y.F. (2016). Simulation Study on the Impact of Tree-Configuration, Planting Pattern and Wind Condition on Street-Canyon’s Micro-Climate and Thermal Comfort. Build. Environ..

[77] Qwaider S., Al-Ramadan B., Shafiullah M., Islam A., Worku M.Y. (2023). GIS-Based Progress Monitoring of SDGs towards Achieving Saudi Vision 2030. Remote Sens..

[78] Hakimdavar R., Hubbard A., Policelli F., Pickens A., Hansen M.C., Fatoyinbo T., Lagomasino D., Pahlevan N., Unninayar S., Kavvada A. (2020). Monitoring Water-Related Ecosystems With Earth Observation Data in Support of Sustainable Development Goal (SDG) 6 Reporting. Remote Sens..

[79] De la Torre S., Morelos-Juárez C. (2022). Primate Conservation Efforts and Sustainable Development Goals in Ecuador, Combining Research, Education and Capacity Building. Animals.

[80] Honeck E., Castello R., Chatenoux B., Richard J.-P., Lehmann A., Giuliani G. (2018). From a Vegetation Index to a Sustainable Development Goal Indicator: Forest Trend Monitoring Using Three Decades of Earth Observations Across Switzerland. ISPRS Int. J. Geoinf..

[81] Edrisi S.A., El-Keblawy A., Abhilash P.C. (2020). Sustainability Analysis of Prosopis Juliflora (Sw.) DC Based Restoration of Degraded Land in North India. Land.

[82] Khaokhrueamuang A. (2014). Sustainability of Rural Land Use Based on an Integrated Tourism Model in Mae Kampong Village, Chiang Mai Province, Thailand. Geogr. Rev. Jpn. Ser. B.

[83] Kotsoni A., Dimelli D., Ragia L. (2017). Land Use Planning for Sustainable Development of Coastal Regions. International Conference on Geographical Information Systems Theory, Applications and Management.

[84] Kalfas D., Kalogiannidis S., Chatzitheodoridis F., Toska E. (2023). Urbanization and Land Use Planning for Achieving the Sustainable Development Goals (SDGs): A Case Study of Greece. Urban Sci..

[85] Cubley E.S., Richer E.E., Baker D.W., Lamson C.G., Hardee T.L., Bledsoe B.P., Kulchawik P.L. (2021). Restoration of Riparian Vegetation on a Mountain River Degraded by Historical Mining and Grazing. River Res. Appl..

[86] Kanga S., Meraj G., Johnson B.A., Singh S.K., PV M.N., Farooq M., Kumar P., Marazi A., Sahu N. (2022). Understanding the Linkage Between Urban Growth and Land Surface Temperature—A Case Study of Bangalore City, India. Remote Sens..

[87] Mahfuza S., Hossain, Islam M. (2021). Impacts of Urbanization on Land Cover Pattern in Bangladesh: A Downscaled Approach for Chuadanga District. J. Environ. Sci. Nat. Resour..

[88] Maghrabi A., Alyamani A., Addas A. (2021). Exploring Pattern of Green Spaces (GSs) and Their Impact on Climatic Change Mitigation and Adaptation Strategies: Evidence from a Saudi Arabian City. Forests.

[89] Abd El Aal A.K., Kamel M., Alyami S.H. (2020). Environmental Analysis of Land Use and Land Change of Najran City: GIS and Remote Sensing. Arab. J. Sci. Eng..

[90] Rahman M., Nahiduzzaman K. (2019). Examining the Walking Accessibility, Willingness, and Travel Conditions of Residents in Saudi Cities. Int. J. Environ. Res. Public Health.

[91] Alnaim A.F., Abdelwahed N.A.A., Soomro B.A. (2022). Environmental Challenges and Green Innovation Strategy: A Vigorous Development of Greener Dynamics. Sustainability.

[92] Alwakid W., Aparicio S., Urbano D. (2021). The Influence of Green Entrepreneurship on Sustainable Development in Saudi Arabia: The Role of Formal Institutions. Int. J. Environ. Res. Public Health.

[93] Almadini A.M., Hassaballa A.A. (2019). Depicting Changes in Land Surface Cover at Al-Hassa Oasis of Saudi Arabia Using Remote Sensing and GIS Techniques. PLoS ONE.

[94] Wasiq M., Kamal M., Ali N. (2023). Factors Influencing Green Innovation Adoption and Its Impact on the Sustainability Performance of Small- and Medium-Sized Enterprises in Saudi Arabia. Sustainability.

[95] Hassan A.H.M., Al Nasser L.A.S. (2023). Analysis of Changes in Coastal Environment Elements and Environmental Impacts in Dammam, Saudi Arabia Using Remote Sensing and GIS. J. King Abdulaziz Univ. Mar. Sci..

[96] Mobarak B., Shrahily R., Mohammad A., Alzandi A.A. (2022). Assessing Green Infrastructures Using GIS and the Multi-Criteria Decision-Making Method: The Case of the Al Baha Region (Saudi Arabia). Forests.

[97] Khan M.Y.A., ElKashouty M., Subyani A.M., Tian F. (2023). Morphometric Determination and Digital Geological Mapping by RS and GIS Techniques in Aseer–Jazan Contact, Southwest Saudi Arabia. Water.

